# Evidence-based goals in LDL-C reduction

**DOI:** 10.1007/s00392-016-1069-7

**Published:** 2017-01-25

**Authors:** Handrean Soran, Ricardo Dent, Paul Durrington

**Affiliations:** 1 Cardiovascular Research Group, School of Biomedicine, University of Manchester, Core Technology Facility, Manchester, UK; 2 Cardiovascular Trials Unit, University Department of Medicine, Central Manchester University Hospitals NHS Foundation Trust, Manchester, UK; 3 Amgen (Europe) GmbH, Zug, Switzerland; 4 Esperion Therapeutics Inc., Ann Arbor, MI USA

**Keywords:** LDL cholesterol, Statin, PCSK9, Ezetimibe, Residual risk

## Abstract

The evidence from trials of statin therapy suggests that benefits in cardiovascular disease (CVD) event reduction are proportional to the magnitude of low-density lipoprotein cholesterol (LDL-C) lowering. The lack of a threshold at which LDL-C lowering is not beneficial, in terms of CVD prevention observed in these trials, is supported by epidemiological and genetic studies reporting the cardio-protective effects of lifelong low exposure to atherogenic cholesterol in a graded fashion. Providing that intensive LDL-C lowering is safe, these observations suggest that many individuals even at current LDL-C treatment targets could benefit. Here, we review recent safety and efficacy data from trials of adjunctive therapy, with LDL-C lowering beyond that achieved by statin therapy, and their potential implications for current guideline targets. Finally, the application of current guidance in the context of pre-treatment LDL-C concentration and deployment of statin therapy is also discussed. The number of patients requiring treatment to prevent a CVD event with statin treatment has been shown to differ markedly according to the pre-treatment LDL-C concentration even when absolute CVD risk is similar. It produces more likelihood of benefit when absolute LDL-C reduction is greater which is largely dependent on pre-treatment LDL-C concentration. This also has to be taken in consideration when deploying new agents like proprotein convertase subtilisin/kexin type 9 monoclonal antibodies. Patients with highest LDL-C concentration despite maximum statin and ezetimibe therapy will attain most absolute LDL-C reduction when treated with proprotein convertase subtilisin/kexin type 9 monoclonal antibodies, hence benefit most in term of CVD risk reduction.

## Introduction

Molecular and cellular studies have established a central role for low-density lipoprotein cholesterol (LDL-C) in the pathogenesis of atherosclerotic plaques, and their clinical sequelae including coronary heart disease (CHD) and ischaemic stroke. Epidemiological data confirm an independent positive association between LDL-C and cardiovascular disease (CVD) risk and suggest that this relationship extends to low LDL-C levels [1, 2]. The significance of lifetime exposure to elevated LDL-C is underscored by genetic studies in individuals with heterozygous familial hypercholesterolaemia (HeFH) who appear to be at considerable excess risk of premature atherogenesis and CVD [3]. Conversely, in populations with genetically determined low LDL-C, CVD risk approximately halves for every 1 mmol/L (39 mg/dL) reduction in LDL-C [4].

Efforts to reduce LDL-C with statin therapy in at-risk individuals have been largely successful in reducing CVD risk, driven largely by a reduction in events rates of myocardial infarction (MI) with no apparent threshold at which LDL-C lowering is not associated with reduced risk [5, 6]. Recent trial data for therapies targeting secreted circulating protease proprotein convertase subtilisin/kexin type 9 (PCSK9) or the Niemann–Pick C1-like 1 (NPC1L1) protein suggest that incremental lowering above and beyond that achieved with statin treatment is possible [7–9]. Furthermore, the use of adjunctive therapy can reduce LDL-C levels below targets recommended by guidelines [10–12]. We evaluate the evidence for residual risk at current LDL-C treatment goals and the implications of recent trial findings for existing guidelines. The safety of LDL-C reductions to very low levels is discussed with reference to genetic, epidemiological and clinical studies.

## Efficacy of LDL-C lowering for CVD event reduction

### Genetic and population data

Identification of LDL-lowering alleles of PCSK9 has allowed the direct effect of very low LDL-C on CVD risk to be determined by Mendelian randomisation, independent of confounding variables such as diet, medical therapy and weight [1, 13]. In 2006, genotyping of 13,342 individuals enrolled in the Atherosclerosis Risk in Communities (ARIC) study provided evidence of protection against CVD in a graded fashion with LDL-C levels, despite a high prevalence of other risk factors [14]. Mutations that lowered LDL-C by around 1 mmol/L (40 mg/dL) reduced incident CHD by around 88%, whereas lowering by around 0.5 mmol/L (20 mg/dL) reduced events by 50%. These effects are bigger than seen in randomised controlled trials (RCTs) of for example statins, emphasising the importance of a lifelong lower LDL-C, over shorter-term reduction later in life.

Mendelian randomisation studies have also demonstrated the importance of lifetime exposure to LDL-C, with genetically determined low LDL-C associated with greater magnitudes of CHD risk reduction as compared with LDL-C lowering to equivalent values in the CTT meta-analyses of statin trials [4]. Table 1 summarises some of the most common conditions with genetically determined low LDL-C.

**Table 1:** Genetically determined causes of low LDL

Monogenic disorder [reference number]	Affected gene	Inheritance	Biochemical features	Adverse features	Long-term outcome
Abetalipoproteinaemia (ABL) [82, 83]	*MTP*	Autosomal recessive	Very low LDL-C	Fat malabsorption, spinocerebellar degeneration, retinitis pigmentosa, acanthocytosis	Reduced life expectancy from non-vascular complications
Homozygous familial hypobetalipoproteinaemia (FHBL) [82, 84]	*APOB*	Autosomal co-dominant	LDL-C <1.3 mmol/L	Fat malabsorption, spinocerebellar degeneration, retinitis pigmentosa, acanthocytosis	Reduced life expectancy from non-vascular complications
Heterozygous familial hypobetalipoproteinaemia (FHBL) [82, 84]	*APOB*	Autosomal co-dominant	LDL-C <5th percentile	Asymptomatic, fatty liver, mild fat malabsorption, diarrhoea	Possible cardioprotective effects of low LDL-C
Primary bile acid malabsorption [85, 86]	*SLC10A2*	Autosomal recessive	Low LDL-C	Diarrhoea, steatorrhoea, failure to thrive	Possible cardioprotective effects of low LDL-C
PCSK9 deficiency (loss of function) [77]	*PCSK9*	Autosomal recessive	Extremely low LDL-C (<1st percentile)	None	88% CVD reduction

*MTP* microsomal triglyceride protein, *APOB* apoprotein B, *PCSK9* protease proprotein convertase subtilisin/kexin type 9, *LDL* low-density lipoprotein, *CVD* cardiovascular disease

### Interventional studies

**Fig. 1:**
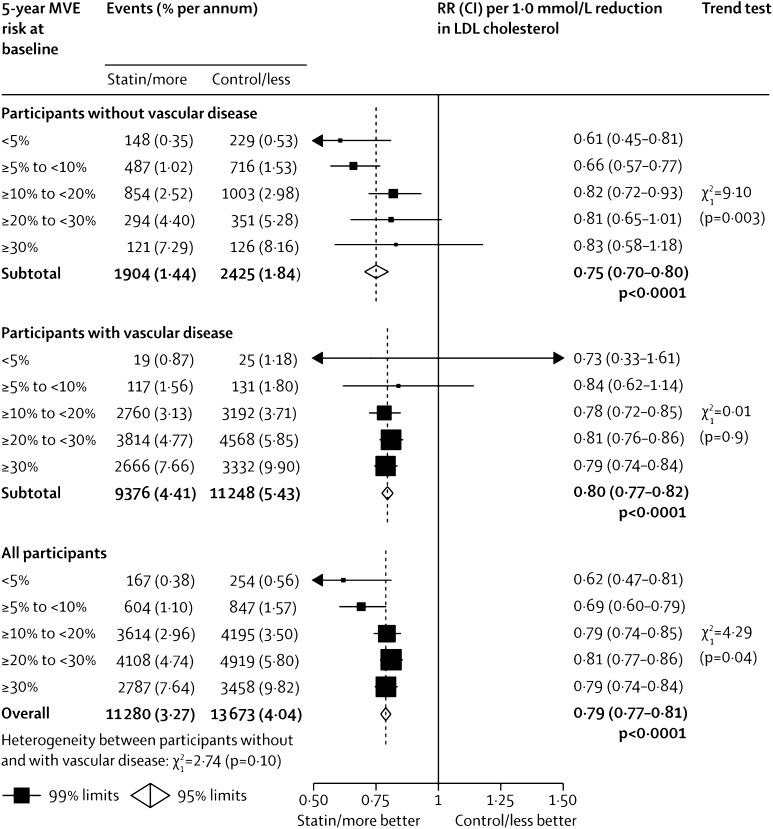
Proportional effects on major vascular events per mmol/L LDL cholesterol reduction. *MVE* major vascular events, *RR* relative risk, *CI* confidence interval. From Baigent et al. [6] with permission of the publisher (Elsevier, 2012)

Taken together, the statin trial data suggest that the absolute benefits of treatment are related to an individual’s risk of atherosclerotic CVD and the absolute reduction in LDL-C that is achieved [15]. Meta-analyses undertaken by the Cholesterol Treatment Trialists’ (CTT) Collaboration on statin trials suggest that a 1.0 mmol/L reduction in LDL-C is associated with a relative risk (RR) of 0.90 (95% CI 0.87–0.93) for all-cause mortality, or in other words, a reduction of 10%. Major coronary events were similarly reduced by 24% (RR 0.76, 95% CI 0.73–0.79) and stroke by 15% (RR 0.85, 95% CI 0.8–0.89) (Fig. 1) [5, 6]. The combined CVD endpoint of CHD plus stroke decreased by 22% (RR 0.78, 95% CI 0.76–0.80) per 1.0 mmol/L reduction in LDL-C [5]. Importantly these benefits were observed irrespective of baseline cholesterol concentration (even with LDL-C <2 mmol/L) and there was no evidence to suggest that achieving low LDL-C levels resulted in any adverse effects. These findings imply that patients at high risk of atherosclerotic occlusive disease should benefit further from achieving the lowest concentrations of LDL-C possible, below even the targets from the US National Cholesterol Education Programme and European Society of Cardiology (ESC) of <2.6 mmol/L [10, 11]. For individuals at very high risk, the European guidelines recommend to achieve both a lower target of <1.8 mmol/L (<70 mg/dL) and an LDL-C reduction from baseline of at least 50%, where baseline LDL-C is between 1.8 and 3.5 mmol/L (70 and 135 mg/dL) [11]. Importantly, these guidelines fundamentally differ from those provided by the American Heart Association (AHA)/American College of Cardiology (ACC) which do not provide a specific treatment targets for LDL-C [16]. The recent ACC expert panel consensus document features two particular developments away from the AHA/ACC 2013 guidelines: the inclusion of specific LDL-C targets and the suggestion to employ adjunctive therapy (ezetimibe and PCSK9 inhibitors) to achieve these targets where maximally tolerated statin monotherapy is insufficient [17]. For patients with pre-existing cardiovascular disease, without comorbidities on statin therapy, the consensus suggests that if the ≥50% reduction in LDL-C or if levels persist above 100 mg/dL, additional LDL-C lowering approaches are warranted. In the case of a patient with pre-existing CVD and LDL-C ≥190 mg/dL, the corresponding LDL-C value at which additional therapy may be considered is 70 mg/dL. This is a significant step toward lowering the threshold for adjunctive treatment and lowering the optimal LDL-C target.

The Improved Reduction of Outcomes: Vytorin Efficacy International Trial (IMPROVE-IT) study enrolled participants with a history of acute coronary syndrome to receive simvastatin and ezetimibe or simvastatin alone [9]. The addition of ezetimibe lowered LDL-C by around 24% and resulted in a significantly lower risk of CV events after 7 years (hazard ratio 0.94, 95% CI 0.89–0.99, *P* = 0.016). The magnitude of benefit seen with additional LDL-C lowering was consistent with that reported in the CTT meta-analyses [5, 6], a finding that lends support to the so-called LDL hypothesis [18]. The absence of benefit in reducing all-cause mortality or deaths from cardiovascular causes in IMPROVE-IT was noteworthy, but not entirely unexpected when considering that prior trials of intensive-dose versus standard-dose statin therapy did not demonstrate a mortality benefit and IMPROVE-IT was not powered to detect a difference in mortality [19].

Recent trials with the PCSK9 inhibitors evolocumab and alirocumab suggest that profound reductions in LDL-C may further reduce CV events despite a limited duration of follow-up [7, 8]. In the two Open-Label Study of Long-Term Evaluation against LDL-C (OSLER) studies [7], participants who had completed one of the twelve phase 2 or 3 studies were assigned in a 2:1 ratio to receive either one of two evolocumab doses (140 mg every 2 weeks or 420 mg monthly) in addition to standard therapy or standard therapy alone. LDL-C was reduced by 61% in the two evolocumab treatment arms compared with standard therapy, equating to a decrease in LDL-C from median baseline levels of 3.95 mmol/L (120 mg/dL) in the parent studies to 1.26 mmol/L (48 mg/dL) in evolocumab groups at week 12 of the open-label studies. Patients in the evolocumab group had a corresponding reduction in CV events as compared with those in the standard treatment group (hazard ratio 0.47, 95% CI 0.28–0.78). Similarly, a post hoc analysis performed on data from the Long-term Safety and Tolerability of Alirocumab in High Cardiovascular Risk Patients with Hypercholesterolemia Not Adequately Controlled with Their Lipid Modifying Therapy (ODYSSEY LONG TERM) study also suggested a beneficial effect of PCSK9 inhibition on major CV events compared with placebo (hazard ratio 0.52, 95% CI 0.31–0.90). ODYSSEY LONG TERM enrolled patients with HeFH and those with at least CHD risk equivalent [8]. Inclusion criteria specified patients on maximally tolerated statin therapy with an LDL-C ≥1.8 mmol/L (70 mg/dL). At baseline, mean LDL-C were 3.2 mmol/L (124 mg/dL). Using alirocumab 150 mg every 2 weeks, mean reductions in LDL-C of 1.2 mmol/L (46 mg/dL) were observed at week 24.

Positive outcomes in OSLER, ODYSSEY and IMPROVE-IT studies are also significant in light of the failure of several other non-statin lipid lowering agents to demonstrate clinical benefit [20, 21]. Further non-statin pharmacological reduction in LDL-C using niacin has recently been investigated in the Atherothrombosis Intervention in Metabolic Syndrome With Low HDL/High Triglycerides and Impact on Global Health Outcomes (AIM-HIGH) study and Heart Protection Study 2–Treatment of HDL to Reduce the Incidence of Vascular Events (HPS2-THRIVE) [22, 23]. Despite previous studies showing a reduction in non-fatal MI with niacin administered as monotherapy to men with raised LDL-C [24], these more recent studies examining the effects of niacin as add-on therapy to statins in patients with pre-existing CVD statin-lowered LDL-C have failed to demonstrate reductions in CVD or all-cause mortality. Given the lowering of LDL-C and triglyceride with niacin, and benefits in raising HDL-C, one may speculate that one of the off-target effects of niacin may attenuate or reverse the clinical benefit that improvement in lipid parameters offers. It is also possible that the increase in HDLs cholesterol target was not associated with an improvement in HDL functionality enough to have an impact [25]. In conjunction with its known side effects of hepatotoxicity, hyperuricemia and hyperglycemia, these outcomes trials have signalled the end of niacin’s use.

Uncertainty exists around the use of another LDL-C lowering strategy, cholesteryl ester transfer protein (CETP) inhibition. Two outcome trials with different CETP inhibitors have failed to show a benefit in terms of CVD risk reduction [21, 26]. The Investigation of Lipid Level Management to Understand its Impact in Atherosclerotic Events (ILLUMINATE) trial enrolled patients with pre-existing CVD or type 2 diabetes and those receiving torcetrapib (CETP inhibitor) in addition to statin experienced a 25% reduction in LDL-C and a 72% increase in HDL-C [26]. Despite this, a significantly increased risk of death in the torcetrapib group led to termination of the trial. The cause for this excess mortality risk is not entirely clear; however, as with niacin it is possible that off-target effects such as raised blood pressure and increased levels of circulating aldosterone, as well as increasing HDL-C without a significant improvement in HDLs functionality may have affected the primary outcome measure [27]. It is interesting to note that post hoc analyses of the trial showed regression of coronary atheroma with greater levels of HDL-C, which was also seen to have an inverse relationship with CVD events. The second outcomes trial using the CETP inhibitor dalcetrapib reported less marked increases HDL-C in the active treatment group (≈30%) with minimal effects on LDL-C and no benefit in reduction of CVD events [21]. Its failure could be explained by the absence of any pre-trial evidence suggesting a stabilising effect on atherosclerotic plaques or the failure to meaningfully reduce LDL-C in the trial [28]. More recent studies demonstrated that the effects of dalcetrapib on atherosclerotic outcomes are determined by polymorphism in adenylate cyclase type 9 gene [29, 30].

## Safety of LDL-C lowering

It is well established that lifelong exposure to elevated concentrations of LDL-C is associated with increased risk of CV morbidity and mortality. There remain, however, some scientific discussions regarding the safety of aggressive LDL-C lowering and the effects of long-term low levels on CV and overall health.

### Clinical trials data

On-treatment LDL-C concentrations below a normal range during extended follow-up in the setting of statin trials do not appear to be associated with any adverse effects [31]. Among patients achieving LDL-C levels of <1.3 mmol/L (50 mg/dL) on rosuvastatin in the JUPITER trial, there were no reported differences in symptoms of myalgia, muscle weakness or myopathy when compared to those who did not meet target. A non-significant increase in diabetes as an adverse event was observed among subjects attaining a target of LDL-C <1.3 mmol/L (50 mg/dL); depression and colon cancer were reported less frequently in this group. In a meta-analysis performed by Sattar et al. evaluating the risk of developing diabetes in patients on statin therapy [32], a 9% increased risk of incident diabetes was reported. Importantly, the small absolute risk for developing diabetes was outweighed by the CVD benefit of statins in the medium term, and change in LDL-C concentration was not associated with increased risk of incident diabetes. Preiss et al. carried out a further meta-analysis of five studies randomising individuals, free of diabetes at baseline, to standard- or intensive-dose statins [33]. Based on a higher risk of developing type 2 diabetes among participants receiving higher-dose statins, the diabetogenic effects of statins were suggested to be dose-related.

The limited follow-up duration of phase 3 trials involving PCSK9 inhibitors precludes a comprehensive assessment of long-term safety; however, the profound lipid lowering achieved offers the opportunity to analyse the occurrence of adverse events with very low LDL-C concentrations over the short term. In a comprehensive analysis of data from the OSLER-1 and OSLER-2 studies [7], the incidence of adverse events including elevation in aminotransferase or creatinine kinase, muscular complaints, and neurocognitive events did not appear to increase among the 26% of patients enrolled achieving LDL-C levels below 0.6 mmol/L (23 mg/dL). These data should be interpreted with caution in light of the short follow-up, more intensive visiting schedule with PCSK9 inhibition therapy versus standard of care and the open-label nature of the OSLER study.

A similar finding was observed in the ODYSSEY LONG TERM study [8]. Over a third of patients in the alirocumab arm had two consecutive LDL-C measurements <0.6 mmol/L (23 mg/dL) and adverse events were similar when compared with the overall treatment group. While not related in either study to the magnitude of LDL-C reduction, neurocognitive defects were reported more often in the group of patients receiving a PCSK9 inhibitor than those on standard of care. Concerns regarding the impact of PCSK9 inhibition on its role in the regulation of neuronal apoptosis has led to demands from the FDA to evaluate possible changes with treatment; however, to date there are no data to support an association with the degree of LDL-C lowering. A dedicated systematic review on the subject of cognitive decline with LDL-C lowering on statin therapy did not support any association [34]. Furthermore, neurocognitive dysfunction has not been reported among individuals with undetectable LDL-C resulting from abetalipoproteinaemia [35], despite frequent problems with absorption of fat soluble vitamins. Vitamin E transport in particular is closely associated with LDL metabolism and severe vitamin E deficiency is characteristic of abetalipoproteinaemia [36]. Substantial LDL-lowering observed in trials of PCSK9 inhibitors was shown to lower absolute vitamin E levels, but not those normalised for cholesterol [37]. While patients with abetalipoproteinaemia are unable to form chylomicrons essential for absorption of vitamin E, those treated with PCSK9 inhibition experience increased catabolism of LDL, which is thought unlikely to impair absorption or distribution of vitamin E. Despite the requirement of free cholesterol for steroid hormone synthesis, no evidence of impairment in adrenal or gonadal steroid hormone synthesis was found with PCSK9 inhibition, even in patients who experienced LDL reductions to <0.4 mmol/L (<15 mg/dL) [37].

### Genetic studies

Familial hypobetalipoproteinaemia (FHBL) is a hereditary disorder of lipoprotein metabolism characterised by very low levels of apolipoprotein B (apoB), and consequently LDL-C. As such FHBL individuals are a good population in which to study the effects and safety of intensive lipid modification therapy. Individuals with heterozygous FHBL are generally asymptomatic although case reports and some small series have suggested that impairment of hepatic very low-density lipoprotein (VLDL)-triglycerides (TGs) secretion may lead to fat accumulation in the liver [38, 39]. Case–control studies have confirmed increased prevalence and severity of hepatic steatosis, as a consequence, among individuals with FHBL [40]. In this study, mean LDL-C concentration among FHBL subjects was 1.04 ± 0.5 mmol/L. Abetalipoproteinaemia and homozygous hypobetalipoproteinaemia are characterised by more profound malabsorption of lipid soluble vitamins leading to retinal degeneration, neuropathy and coagulopathy [36]. Lipid profiles with these disorders would show nearly absent LDL (<0.1 mmol/L) and apoB (<0.1 g/L). Non-sense mutations in PCSK9 are also associated with low plasma levels of LDL-C and apoB, but in heterozygotes, the levels are not as low as in abetalipoproteinaemia and homozygous hypobetalipoproteinaemia and no systemic involvement is seen [36].

Non-sense mutations in PCSK9 are associated with around a 30% reduction in plasma LDL-C levels and confer an 88% reduction in CHD events with no apparent adverse effects [14, 41]. In addition to its role in the study of cardioprotection with PCSK9 variants, Mendelian randomisation can also be used to examine the safety messages of genetically determined low plasma LDL-C levels. A study by Folsom et al. using data from ARIC investigated whether any association existed between PCSK9 variants and cancer [42]. The rationale for this study was based on observations from historical epidemiological studies that identified a modest association between low plasma cholesterol levels and cancer incidence [43, 44]. The mechanism for this association was not clear and several reports from the 1990s suggested it was likely to be an example of reverse causality, where cancer could result in low plasma LDL-C and total cholesterol [43, 45]. The ARIC analysis found that lifelong low cholesterol concentration, as reflected by PCSK9 variants in black and white individuals, was not associated with increased risk of cancer.

## Residual risk on statin therapy

Meta-analyses of statin trials provide evidence of significant on-treatment residual risk, with 5-year major CV event rates of 22% among individuals with prior CVD and 10% among those without prior disease [5, 46]. Pooled data support the concept of LDL-C as a marker of residual risk, even with LDL-C levels <2 mmol/L (<77 mg/dL) [5, 6]. Among patients with stable coronary disease in the Treating to New Targets (TNT) trial, 8.7% of patients receiving atorvastatin 80 mg with on-treatment LDL-C concentrations of 1.8–2.6 mmol/L (70–100 mg/dL) experienced a major event over 5 years [47]. The harbingers of this residual risk can be classified into lipid-related and non-lipid factors. In terms of the lipid-related factors, HDL cholesterol and TGs are commonly implicated [48]; however, the evidence to support an independent association of either of these parameters with major CV events is inconsistent irrespective of adjustment for apolipoproteins [49–53]. Similarly, while on-treatment LDL-C was predictive of outcome in JUPITER [54], this finding was not replicated in other studies after adjustment was made for apolipoproteins and other clinical risk factors [55]. It is noteworthy that statins can modify the plasma concentrations of most atherogenic lipids and isolating the influence of individual lipid parameters on cardiovascular risk is complicated.

A significant proportion of selected populations in secondary prevention trials with high-dose statin do not obtain optimal LDL-C levels, despite close monitoring of adherence that is not feasible in clinical practice. As such, the removal of specific lipid goals in the 2013 American College of Cardiology (ACC)/American Heart Association (AHA) guidelines [16], leaves little scope for residual risk assessment when response to treatment is inadequate. Accordingly, there is no support in the guidance for the use of additional lipid-lowering therapies among those with high absolute risk who are on maximal statin therapy or who have achieved a 50% reduction in LDL-C. This leaves an important modifiable risk factor, high LDL-C despite statin therapy, not monitored neither addressed in many patients.

## Deployment of statin therapy and side effects

In 2007 the National Institute for Health and Care Excellence (NICE) issued guidance recommending statin therapy for primary prevention in individuals with a ≥20% 10-year risk of developing CVD [12]. It resulted in a significant increase in statin therapy for high-risk individuals, with UK population data suggesting treatment increased from 7.0% prior to 2007 to 30.4% from 2007 onwards [56]. More recent guidance documents from NICE have advocated even a lower threshold for statin treatment, at 10% 10-year risk CVD risk [57]. The perceived medicalization of a growing number of patients being offered statin therapy as a result of the lower threshold for treatment has also placed a significant burden on primary care providers [58].

The side effects associated with statin use are well documented in the medical literature and lay press [59]. However; adverse effects on standard doses of statin therapy are unusual; the incidence rate of myopathy is thought to be around one patient in 10,000 person years on treatment [60], and the adverse effects are reversed following discontinuation of statins. Furthermore, the majority of muscle symptoms that occurs on-treatment with statins are not thought to be attributable to statins themselves [60]. Liver disease as a result of statin therapy is also rare, meta-analysis of three randomised trials of pravastatin showed that hepatobiliary disorders were less frequently recorded in groups on statin as compared with placebo. Among the strongest signal for a side-effect associated with statin therapy is peripheral neuropathy which is thought to occur at a rate of around 1 per 10,000 person years on treatment [60]. What remains irreversible is the significant morbidity and mortality associated with atherosclerotic CVD. A recent concerning report suggests that negative news stories concerning side effects with statin therapy have implications for adherence on a population level and cause significant harm in terms of preventable CVD events and death [61].

The adoption of a more targeted approach to identify individuals who will benefit most from statin therapy by considering pre-treatment cholesterol values as well as absolute CVD risk has been suggested [62]. The number of patients requiring treatment to prevent a CVD event with statin treatment has been shown to differ markedly according to the pre-treatment LDL-C concentration even when absolute CVD risk is similar [63]. It produces more likelihood of benefit when pre-treatment LDL-C concentration is initially high because the reduction in LDL-C is greater. Atorvastatin 80 mg daily (recommended by both NICE and the ACC/AHA for high-risk patients such as those who have already experienced a CVD event) produces a 55% decrease in LDL-C [12, 55]. Thus, in individuals with an initial LDL-C of 2.5 mmol/L, atorvastatin 80 mg daily will typically reduce LDL-C to 1.125 mmol/L, whereas an estimated reduction to 2.5 mmol/L would be anticipated in a patient with a pre-treatment LDL-C of 5.5 mmol/L. It may come as a surprise to many healthcare professionals that some opinion leaders are advocating LDL-C lowering to levels as low as or lower than 1.125 mmol/L; however, these calls are based on the evidence reviewed in this article and are a rational translation of the evidence into clinical practice. The present author’s opinion is that it remains highly irrational to have this low target restricted to individuals with low pre-treatment LDL-C concentration. In such cases, there is a greater likelihood that an incident CVD event would be attributable to risk factors other than LDL-C. If the current guideline algorithm from NICE is followed, a patient with a higher pre-treatment LDL-C may remain with an LDL-C level at 2.5 mmol/L and not receive adjunctive therapy to achieve a similar LDL-C (1.125 mmol/L) to that of a patient with a starting value of 2.5 mmol/L. Table 2 shows that when the risk of a further CVD event is say 40% over the next 10 years, more events are prevented in patients with a higher pre-treatment LDL-C, and even more when greater LDL-C reductions are achieved in patients with high starting values.

**Table 2:** Prevention of cardiovascular disease events according to pre- and on-treatment LDL-C levels among individuals at high risk of CVD

	Case study 1	Case study 2
10-year CVD risk	40%	40%
Pre-treatment LDL-C	2.5 mmol/L (96.7 mg/dL)	5.5 mmol/L (212.7 mg/dL)
LDL-C on atorvastatin 80 mg daily	1.125 mmol/L (43.5 mg/dL)	2.5 mmol/L (96.7 mg/dL)
CVD events prevented per 100 patients treated with atorvastatin 80 mg daily^a^	12	21
CVD events prevented per 100 patients treated when LDL-C reduced to 1.125 mmol/L with atorvastatin 80 mg plus adjunctive treatment (if necessary)^a^	12	27

*CVD* cardiovascular disease, *LDL-C* low-density lipoprotein cholesterol

^a^Based on risk ratio of 0.78 for cardiovascular disease event associated with each 1 mmol/L reduction in LDL-C [5]. Calculation: CVD events prevented/100 patients treated = 40(1 − 0.78^LDL-C reduction^) [62, 63]

## Achievement of LDL-C targets

Many questions about the dissemination of cholesterol guidelines and their impact on practice remain; the magnitude of benefit in terms of LDL-C reduction and subsequent risk reduction observed in clinical trials on which guidelines are based has not been translated on a population-level. Patients with established CHD have substantial absolute risk and are therefore considered a priority for secondary prevention. Numerous studies based on population data report systemic under-utilisation of lipid lowering therapies and failure to meet targets. In an unselected cohort of patients with CHD in German primary care, just 24% achieved contemporary goals of LDL-C <2.59 mmol/L (100 mg/dL) [64]. Registry data of patients with established CVD and/or diabetes enrolled to the Vascular Protection (VP) and Guidelines Orientated Approach to Lipid Lowering (GOALL) study suggest goal attainment in around half of patients, although this figure was reduced to 21% among patients at the highest overall risk [65].

Qualitative review of goal attainment in at-risk patients supports the notion that individuals at the highest absolute risk appear to be least likely to achieve lipid goals [66]. In the GOALL registry, poor adherence to treatment guidelines was not attributed to lack of physician awareness of treatment guidelines. Instead, results suggested that poor patient adherence, inappropriate drug or dose selection, and limited efficacy, were all barriers to successful implementation of contemporary guidance. In the PHARMO database from the Netherlands, over half of 59,094 individuals, each followed for the first two treatment years, discontinued statin therapy. A 30% reduction in the risk of hospitalisation for MI was observed among those who persisted with statin therapy during the follow-up period [67].

## Merits of a targeted approach to LDL-C

On-treatment monitoring and targets are an important and familiar aspect of clinical practice, and can aid communication between doctor and patient, while improving patient compliance. Targets in well-defined treatment paradigms are well established in hypertension, for example, and provide primary care physicians with clear and user-friendly tools to monitor response to treatment. The move away from specific LDL-C targets in the 2013 ACC and AHA guidelines was met with considerable debate [55, 68, 69]. One reason for the marked divergence from pre-existing guidelines [70], was the remit given to the ACC/AHA panel, which limited the evidence considered to RCTs and their strict inclusion criteria, i.e., the absence of a target strategy in RCTs precludes any recommendations for specific targets. Despite this approach, clinical decisions are often required in the absence of RCT data, and the National Lipid Association suggest that treatment goals are a useful strategy to ensure that the intensity of therapy to lower atherogenic cholesterol is matched to absolute risk for a CVD event [71]. A further consequence of the absence of specific treatment targets is the potential to create barriers for access and reimbursement. These barriers are specific to individual healthcare systems but will likely only result in under-utilisation of effective treatments.

The strongest argument for a return to goal strategy exists for those with the highest overall risk and high pre-treatment LDL-C [60]. Residual risk among patients in this group who fail to achieve significant LDL-C reductions could be ignored with an unmonitored approach, as the potential for ‘fire and forget’ is ominous. In a recent patient-level meta-analysis of statin trials, 1-year major CV event rates were 4.4, 10.9 and 16.0% among individuals achieving LDL-C concentrations <1.3 mmol/L, between 1.3 and 1.8 mmol/L, and between 1.8 and 2.6, respectively [72]. This is consistent with IMPROVE-IT results. These findings, in addition to early clinical benefit seen with more dramatic reductions in LDL-C to levels around 1.2 mmol/L (48 mg/dL) with PCSK9 inhibitors in OSLER and ODYSSEY LONG TERM, will rekindle arguments for a return to goal-directed therapy. However, based on the previous approach taken by the ACC/AHA, this shift in emphasis will require evidence from RCTs with a targeting strategy.

One group of patients in whom a move away from targets may be beneficial is patients with high overall risk in the absence of high LDL-C. These patients are likely to have residual risk attributable to LDL-C that is potentially modifiable by achieving lower LDL-C levels therapeutically than was previously recommended (previous target was <1.8 mmol/L in very high risk). With the recommended fixed dose statin treatment these patients will therefore already be able to achieve and benefit from reduction in LDL-C to levels well below 1.8 mmol/L, if they have initially low levels of LDL-C. Atorvastatin in doses of 20 mg daily is recommended for primary prevention, which typically reduces LDL-C by 43%, and in doses of 80 mg daily for secondary prevention, which decreases LDL-C by on average 55% [57, 73]. Thus, in primary prevention people with LDL-C levels <3.16 mmol/L will generally reach values below 1.8 mmol/L and in secondary prevention patients with initial LDL-C concentrations <4 mmol/L will achieve lower levels than 1.8 mmol/L. Paradoxically, however, those patients with higher initial LDL-C who are often more at risk and could receive higher doses of atorvastatin in primary prevention or adjunctive therapy in secondary prevention to allow them to reach lower LDL-C targets below 1.8 mmol/L will be left with suboptimal reductions in LDL-C, in many cases well above the earlier target of 1.8 mmol/L.

Individuals with FH represent a particularly high-risk group in whom premature CHD manifests as a result of lifelong elevated LDL-C. ESC/EAS guidelines suggest an LDL-C target of <2.5 mmol/L in adults with FH [70]. Despite use of high-intensity statins, many adults with FH will not achieve this. Other therapies used in combination with statins (or as monotherapy for individuals unable to tolerate statins) include ezetimibe, Colesevelam and PCSK9 inhibitors. The European Medicines Agency has approved the use of the PCSK9 inhibitors, evolocumab and alirocumab, in adults with heterozygous familial or the non-familial form of primary hypercholesterolaemia or with mixed dyslipidaemia [74, 75]. Evolocumab is also authorised in Europe for use in homozygous familial hypercholesterolaemia. In the UK, the NICE appraisal of this new class of lipid modification therapy is ongoing [76]. Randomised trials of PCSK9 inhibitors evolocumab and alirocumab in FH individuals on existing lipid lowering therapy reported LDL-C reductions of 70 and 67%, respectively [77, 78]. The results of two large cardiovascular outcomes trials randomising patients on statin therapy to receive PCSK9 inhibition or placebo are awaited. The FOURIER trial with evolocumab has enrolled 27,500 patients with a history of MI, ischaemic stroke or symptomatic peripheral artery disease and LDL-C ≥1.8 mmol/L (70 mg/dL) or non-HDL-C ≥2.6 mmol/L (100 mg/dL) [79]. The ODYSSEY Outcomes trial with alirocumab differs in its inclusion criteria, and is expected to randomise around 18,000 participants within 12 months of a hospitalisation for acute MI or unstable angina [80]. Patients must fulfil either the same LDL-C or non-HDL-C criteria as in FOURIER, or have an apolipoprotein B ≥2.1 mmol/L (80 mg/dL).

## Conclusions

Recent trial data evaluating combination therapy among individuals at high risk of CVD suggests that incremental LDL-C lowering, beyond that achieved by statin therapy can translate into reductions in CVD event rates. The evidence from these studies, and that from meta-analyses of statin trials, suggests that the risk to benefit ratio remains positive without a minimum threshold. As long-term data to directly assess the benefit of novel treatments in CVD prevention emerge, it is critical that the deployment of established statin therapy is optimised to achieve its potential. For optimum clinical effectiveness, initial LDL-C concentration must be considered in deciding whether a target will allow a greater decrease in LDL-C and a lower NNT than a fixed dose regimen. Treatment targets will produce greater benefit for patients with high pre-treatment LDL values, but will often mean that people with lower initial levels, if treated to a target LDL-C with the low doses of statin required for this, will not receive the benefit that they might from higher fixed dose statin treatment. Individual variation in the LDL-C response to statins also makes post-treatment cholesterol measurement essential [81]. The same principle should be followed when new agents like PCSK9 monoclonal antibodies are considered.
